# Changes in Patient Safety Culture Alongside Leadership WalkRounds in Shaqra General Hospital from 2023 to 2025

**DOI:** 10.3390/healthcare14213704

**Published:** 2026-11-01

**Authors:** Mahah Saad Almohanna, Abeer Ghazi Alharbi

**Affiliations:** 1 Quality and Patient Safety, Shaqra General Hospital, Riyadh Third Health Cluster, Shaqra 15557, Saudi Arabia; 2 Health Administration, College of Business Administration, King Saud University, Riyadh 11451, Saudi Arabia

**Keywords:** patient safety culture, leadership walkrounds, benchmarking, incident reporting, organizational learning, hospital safety, Saudi Arabia

## Abstract

**Highlights:**

**What are the main findings?**

- Nine of ten patient safety culture domains improved after covariate adjustment and correction for multiple testing, including hospital management and supervisor support.
- Survey participation increased from 36.73% to 67.77%; national benchmark comparisons were descriptive.

**What are the implications of the main findings?**

- Communication, leadership responsiveness, and follow-up are practical improvement priorities for hospitals working within existing resources.
- These locally actionable practices deserve attention alongside structural workforce measures, as Staffing and Work Pace remained the lowest-scoring domain.

**Abstract:**

Background/Objectives: This study examined changes in patient safety culture (PSC) alongside Patient Safety Leadership WalkRounds in a Saudi secondary hospital. Methods: In this retrospective repeated cross-sectional study, anonymous surveys from August 2023 and August 2025 were compared using percent-positive scores and respondent-level regression adjusted for job group, hospital tenure, working hours, and patient contact. Sensitivity analysis reweighted the 2025 role distribution to match that of 2023. National comparisons were descriptive. WalkRounds records from January–June 2025, coded follow-up actions, and annual occurrence variance report (OVR) totals described implementation context. Results: Participation increased from 180/490 (36.73%) to 349/515 (67.77%). Overall percent positive increased from 69.43% to 78.50% (difference 9.07 percentage points; bootstrap 95% confidence interval (CI) 5.45–12.85). The adjusted overall score difference was 0.243 (95% CI 0.149–0.337; *p* < 0.001). Nine domain differences remained significant after Holm adjustment, including hospital management and supervisor support; Staffing and Work Pace remained the lowest-scoring domain and its change was inconclusive (adjusted difference 0.109; 95% CI −0.016–0.234). The ledger documented 27 rounds, 106 staff participations, and 140 issues. Among 51 eligible follow-up actions, 12 (23.5%) were classified as strong, 32 (62.7%) as intermediate, and 7 (13.7%) as weak. Annual OVR counts increased from 1,633 to 1,954, while the proportion of survey respondents reporting no events increased from 26.7% to 35.2%. Conclusions: PSC ratings, including leadership support, were more favorable in 2025. Communication and leadership follow-up are practical priorities alongside structural workforce needs. WalkRounds’ contribution was not isolated, and reporting indicators were mixed.

## 1. Introduction

Patient safety remains a central priority in healthcare because preventable harm continues despite advances in clinical knowledge, technology, and regulation. The World Health Organization frames patient safety as a systems issue that depends not only on technical competence but also on the organizational conditions that enable safe care, including leadership, communication, work processes, and learning systems [1]. Within this perspective, patient safety culture (PSC) has become an important dimension of healthcare quality because it reflects the shared values, norms, and behaviors that shape how healthcare workers recognize risk, speak up about concerns, report incidents, respond to error, and learn from everyday practice [2]. Stronger safety cultures have been associated with better safety processes and more favorable quality-related outcomes, making PSC an important target for measurement and improvement in hospitals [3,4].

In Saudi Arabia, PSC remains an important leadership concern. A systematic review and recent Saudi studies have shown persistent variation across hospitals, with recurring weaknesses in management support for patient safety, response to error, staffing and work pace, and reporting culture, despite relatively stronger performance in teamwork and organizational learning in some settings [5,6,7,8,9]. In a recent national Saudi study, 48.3% of healthcare practitioners reported no events in the previous 12 months, and 25.1% reported only one or two events [9]. These findings highlight the importance of organizational conditions that support reporting, speaking up, and learning from concerns.

Standardized PSC surveys help hospital leaders identify priorities, compare domains, and track staff perceptions over successive cycles. The Agency for Healthcare Research and Quality (AHRQ) Surveys on Patient Safety Culture (SOPS) program provides a framework for repeated assessment and benchmarking [10]. Studies of earlier versions of the Hospital Survey on Patient Safety Culture demonstrate the importance of examining local measurement properties when interpreting domain scores [11]. For hospital management, the next step is to translate survey findings into organizational action. Reviews of PSC interventions emphasize the design and implementation of improvement programs, including leadership engagement and structured work with frontline teams [12,13].

Patient Safety Leadership WalkRounds (PSLWR) put leadership engagement into practice through structured discussions with frontline staff in their care areas [14]. These discussions give leaders direct access to concerns about daily work and create opportunities to identify hazards and agree on responses. Prior studies have reported associations between WalkRounds participation, safety climate, and workforce outcomes, with feedback on actions taken being a prominent feature of favorable findings [15,16,17]. Follow-up therefore deserves explicit attention in evaluations of WalkRounds. A systems approach examines whether responses address the organizational conditions that generate recurrent hazards [18]. The Institute for Healthcare Improvement (IHI) action hierarchy supports this assessment by distinguishing stronger system changes from measures that depend mainly on staff memory, training, or adherence [19]. Examining action strength adds information about the design of the organizational response to the issues raised.

In Saudi Arabia, the Saudi Patient Safety Center (SPSC) national PSC program provides facility reports and national reference values for participating hospitals [20]. Hospitals can use these reports alongside local records of leadership engagement and follow-up to examine their improvement priorities. For secondary hospitals, evaluating these routinely collected data can inform decisions about communication, work organization, and responses to staff-identified concerns.

WalkRounds evaluations need to describe the organizational responses that accompany leadership engagement. A systematic review identified associations with operational and perceived cultural outcomes and called for further evidence on clinical outcomes [21]. Evaluations in other health-system settings can extend this evidence by combining repeated PSC assessment with WalkRounds records and action-hierarchy coding. Applying this approach in a Saudi secondary hospital provides context-specific evidence on staff perceptions, the concerns raised, and the types of responses recorded to inform leadership follow-up and improvement priorities.

This study aimed to evaluate changes in PSC at Shaqra General Hospital between August 2023 and August 2025, and to characterize documented PSLWR activity and the strength of follow-up actions. National benchmarks and annual occurrence variance report (OVR) totals provided context for the evaluation.

## 2. Materials and Methods

### 2.1. Study Design

This study used a retrospective, facility-level observational design with a repeated cross-sectional comparison of anonymous PSC surveys conducted in August 2023 and August 2025. Routinely collected WalkRounds records and follow-up actions were used to describe leadership engagement and organizational responses during the period between the surveys.

### 2.2. Setting

Shaqra General Hospital is a secondary general hospital in Riyadh Third Health Cluster, Saudi Arabia, with approximately 150 beds. Staff counts reported for the SPSC facility surveys were 490 in 2023 and 515 in 2025, an increase of 25 (5.1%) in the reported denominator. These counts were compiled from Human Resources using the staff categories specified for facility reporting and cross-checked with departments; they may not represent a complete census of all hospital personnel. The hospital director remained the same between the surveys. During this interval, the hospital underwent an accreditation survey and received accreditation in March 2024. Both PSC surveys were conducted in August. The 2023 and 2025 surveys were consecutive national program cycles (Cycles 5 and 6); no SPSC survey was conducted in 2024 [20]. Formal hospital-level WalkRounds began in January 2025, alongside existing departmental rounds and leadership meetings. The analyzed ledger covers January–June 2025, before the August survey. A concurrent hospital risk-and-harm reduction initiative operated during 2024–2025 and involved incident review, corrective-action follow-up, and feedback. This initiative was not evaluated separately in the study. WalkRounds and concurrent local improvement initiatives were implemented without a dedicated budget, using existing hospital resources. Additional staff recruitment depended on Ministry-level decisions outside the hospital’s authority.

**Figure 1:**
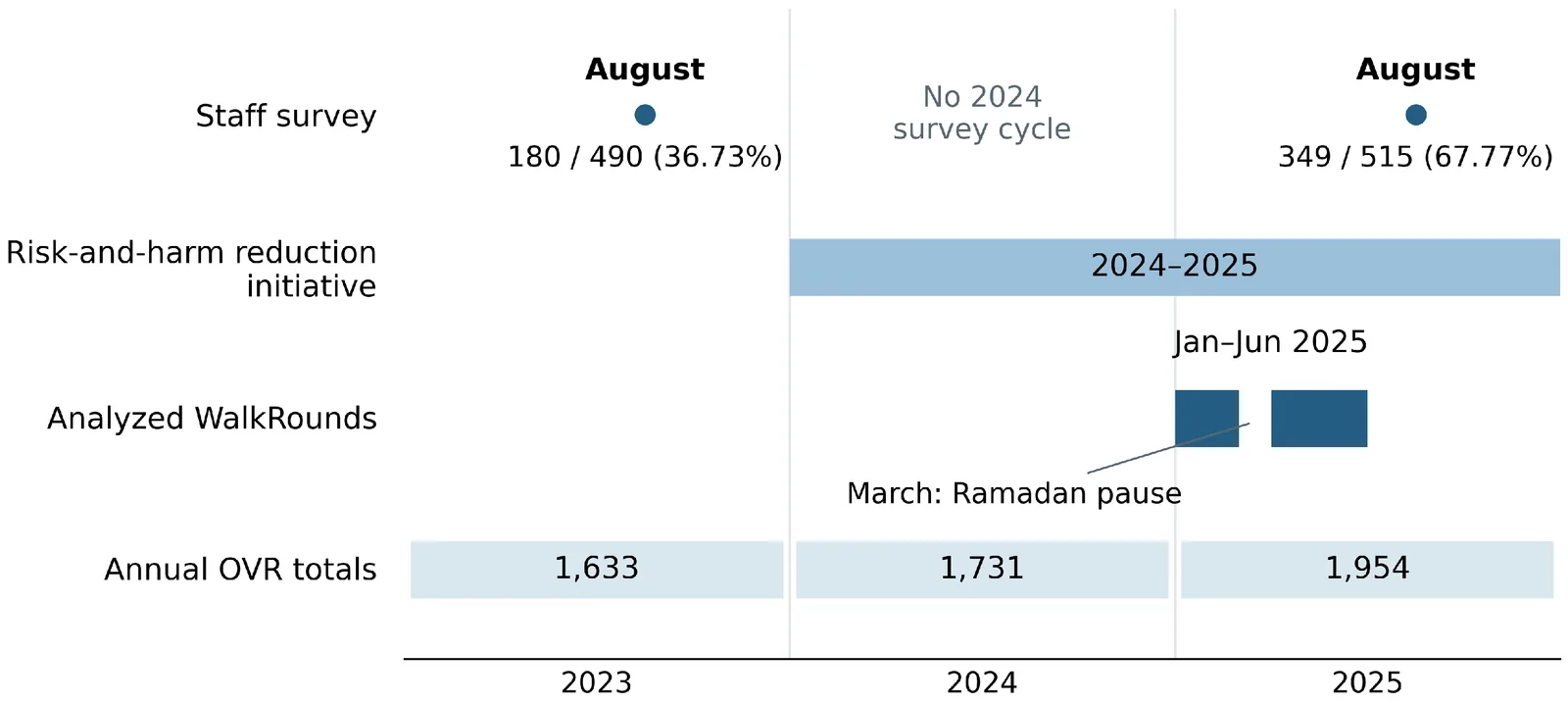
Timing of the survey cycles and organizational activities. Survey labels give completed responses divided by the reported staff denominators. Vertical lines mark calendar-year boundaries, and the leader line identifies the March pause. The concurrent initiative is shown by calendar year as context; its outcomes were not evaluated. The WalkRounds bar covers the analyzed January–June 2025 records; March rounds were paused for Ramadan. Annual occurrence variance report (OVR) totals cover calendar years and do not match the surveys’ preceding-12-month recall periods.

Recognition in 2023–2024 focused on staff who reported near misses. During the 2025 WalkRounds, staff were informed about cluster-level recognition for the most frequent reporters; recognition took place in September 2025, after the August survey. Figure 1 summarizes the survey dates, the concurrent improvement initiative, the analyzed WalkRounds period, and the annual OVR reporting windows.

### 2.3. Data Sources, Study Population, and Measures

Five organizational data sources were used: SPSC facility reports and raw survey responses for the two available cycles; same-year national reference values from the SPSC portal; PSLWR logs and dashboards for January–June 2025; the coded follow-up action dataset; and annual OVR totals. These sources described PSC outcomes, national reference comparisons, WalkRounds coverage and concerns, action themes and hierarchy levels, and incident-reporting volume, respectively.

All completed survey responses available in the raw datasets were included: 180 in 2023 and 349 in 2025. In both cycles, questionnaires were distributed through employee email channels to all staff across all departments and units, with Arabic and English versions available. An “Other” option was available for staff classification in the questionnaire. In 2023, additional departmental visits were needed to clarify the survey’s purpose, whereas staff appeared more familiar with its purpose in 2025. No formal safety-culture or survey-purpose training was provided. Responses were fully anonymous and could not be linked across waves. Survey work-area labels did not identify individual participation in rounds or establish a verified unexposed comparison group.

The SPSC-administered survey used the 32 scored items and ten domains of AHRQ SOPS Hospital Survey version 2.0 [10,20]. The scored item structure was the same in both raw survey files. The ten domains were Teamwork; Organizational Learning—Continuous Improvement; Supervisor/Manager/Clinical Leader Support for Patient Safety; Communication about Error; Communication Openness; Reporting Patient Safety Events; Handoffs and Information Exchange; Hospital Management Support for Patient Safety; Response to Error; and Staffing and Work Pace. Hospital recommendation, patient safety grade, and self-reported number of events in the previous 12 months were also summarized. The SPSC program describes using the official AHRQ survey in Arabic and English [20]. No new translation or adaptation was undertaken for this secondary analysis. A separate record detailing SPSC translation or adaptation procedures was not available, so equivalence of language versions was not established in this evaluation.

The hospital’s operational WalkRounds form included four ratings on a 1–5 scale. Participating frontline staff agreed on one score for each rating during the round, with leaders present. No separate psychometric validation of this form was available. These ratings were summarized as round-level group observations.

The local guideline assigned WalkRounds to hospital, medical, nursing, and quality/patient safety leadership across clinical and support areas. The ledger provides the observed coverage; random selection or a formal risk-based area-selection procedure was not documented. Form prompts covered safety concerns, barriers to safe practice and incident reporting, reporting confidence, leadership involvement, improvements witnessed, and involvement in quality improvement. During rounds, staff were asked about their understanding of the OVR submission process and received explanations where needed. Discussions clarified the role of OVRs in learning and improvement, including addressing the misconception that a report was a complaint against a colleague.

As the hospital’s Patient Safety Coordinator, the first author developed the local WalkRounds guideline and departmental plan, coordinated scheduling, accompanied leaders, documented findings and corrective plans, followed action closure, and prepared the study data. Descriptions of survey dissemination and implementation context also drew on the first author’s operational experience. Actions and follow-up were communicated by email to department heads. Closure was verified using supporting documents or onsite checks. Receipt of feedback by every frontline staff member was not measured.

The patient safety department classified follow-up actions by one primary theme and a strong, intermediate, or weak hierarchy level using the IHI Action Hierarchy Tool, part of Root Cause Analyses and Actions (RCA^2^) and derived from the US Department of Veterans Affairs (VA) National Center for Patient Safety hierarchy [19]. Systems-oriented analysis and intervention-strength research provide context for interpretation [18,22]. The analysis used the department’s recorded classifications, which both authors reviewed and agreed upon; independent duplicate coding and formal adjudication were not documented, so inter-rater agreement could not be reported.

The WalkRounds reports contained 163 extracted candidate recommendation units. The department’s coded follow-up dataset comprised 51 distinct, explicit action units assignable to a primary theme and hierarchy level. The remaining units were excluded if they (1) duplicated or overlapped with another action; (2) consisted of lessons learned, previously resolved problems, or rationale without a specified corrective action; (3) involved escalation to higher authorities for action that could not be implemented within the hospital; or (4) involved follow-up or compliance monitoring of an existing policy, protocol, or practice without a new corrective action. Reason-specific counts are provided in Table S1. The 140 safety issues were counted separately from recommendation units, because one issue could generate more than one recommendation.

### 2.4. Data Analysis

Raw survey responses were cross-checked against the official facility reports. Items were scored using SOPS guidance [10,23]. Negatively worded items were reverse-coded so that higher 1–5 scores were more favorable. Positive responses were scored values of 4 or 5, equivalent to original responses of 1 or 2 for negatively worded items. Missing and “Does not apply/Don’t know” responses were excluded from item denominators.

Respondent-level domain scores were the mean of available items, requiring at least one scored item. An exploratory overall summary was calculated as the equal-weight mean of available domain scores; this is not a validated unidimensional AHRQ total score. Item percent-positive values were computed using valid item denominators, domain percentages were the unweighted mean of their constituent item percentages, and the overall percent-positive value was the unweighted mean of the ten domain percentages.

National reference percentages were taken without recalculation from the “Database Avg. Positive Response” field in the SPSC Hospital Portal Standard Facility Reports for Cycle 5 (2023) and Cycle 6 (2025) [20]. Gaps were calculated as hospital minus same-year national percentages. The difference between hospital and national changes was interpreted descriptively; the national database was not a matched or untreated control group, and two waves did not permit assessment of parallel trends [24]. National hospital and respondent counts, hospital overlap across cycles, Shaqra’s inclusion, and the pooling or averaging method were unavailable to the investigators. National respondent-level data and standard errors were also unavailable, so no confidence intervals or significance tests were calculated for national comparisons.

WalkRounds outputs, themes, and recorded hierarchy levels were summarized descriptively. Available OVR data consisted of annual aggregate counts. Admissions, patient-days, and event-level severity data were unavailable for this analysis; activity-standardized rates and patient-harm outcomes were therefore not estimated. Calendar-year OVR totals did not match the surveys’ preceding-12-month recall periods, and the 2025 total included months after the August survey.

Unadjusted differences between the August 2023 and August 2025 survey waves were estimated using Welch tests and 95% confidence intervals. Linear regression models estimated the difference associated with survey year, adjusting for functional job group, hospital tenure, weekly working hours, and direct patient contact. Covariates were categorical. Twenty-four possible job-position codes were mapped to eight functional groups to limit sparse model cells. No outcome-based covariate selection was used. Heteroskedasticity-consistent HC3 standard errors and residual-degree-of-freedom t intervals were used. Models included all respondents with an observed outcome; adjustment covariates were complete. Ten domain-level *p* values were adjusted using Holm’s method. The overall summary was assessed separately. Confidence intervals are pointwise, not simultaneous intervals.

For the adjusted-score comparison, adjusted scores for both years were obtained by averaging model predictions over the same pooled covariate distribution among respondents with an observed score for each outcome. Pointwise 95% confidence intervals used linear contrasts of fitted coefficients, HC3 covariance estimates, and residual-degree-of-freedom t critical values, conditional on the observed covariate distribution. Adjusted differences equal the corresponding survey-year regression coefficients. Domains are ordered by these point estimates; no formal comparisons between domain changes were performed.

For hospital percent-positive differences, 95% percentile bootstrap confidence intervals were calculated using 10,000 independent resamples of respondents within each wave, retaining each respondent’s item responses together. These intervals describe uncertainty under the assumption of independent responding samples and do not correct nonresponse bias.

Sensitivity analysis reweighted 2025 respondents to the observed 2023 distribution of exact job-position categories. Reweighting used the ratio of the 2023 to 2025 observed role proportions, leaving 2023 weights at one. Weighted year-only regression used HC3 intervals conditional on the estimated weights; roles absent in 2023 received zero weight in 2025. This analysis was performed for the overall summary and all ten domains, with Holm correction across the ten domain tests. Additional sensitivity analyses of the overall summary involved having at least half the items within each domain before scoring; restricting analysis to respondents with all 32 items scored; or additionally adjusting for unit tenure and survey language.

The ordered distribution of self-reported events was compared using a two-sided Mann–Whitney test with tie correction. An exploratory logistic model for reporting no events versus any events adjusted for the same covariates as the primary linear model and used sandwich standard errors. As a descriptive subgroup check, nursing respondents reporting no events were cross-tabulated by hospital tenure of less than one year versus at least one year in each wave. Cronbach’s alpha was calculated using complete cases within each domain; the two-item Reporting Patient Safety Events domain was additionally described using its inter-item correlation and Spearman–Brown coefficient.

Analyses were conducted using Python 3.12.14 (Python Software Foundation, Wilmington, DE, USA) and the community-developed open-source libraries NumPy 2.3.5, pandas 2.2.3, and SciPy 1.17.0. Survey waves were treated as independent because anonymous responses could not be linked; repeat participation may therefore affect uncertainty estimates.

### 2.5. Ethical Considerations

This study used anonymous staff-survey responses and de-identified organizational records, including WalkRounds documentation, coded follow-up actions, and aggregate incident-reporting totals. No patient identifiers were used, and no patients or staff were contacted for research purposes. Files were handled under institutional confidentiality and data security requirements. The Institutional Review Board of Riyadh Third Health Cluster, Ministry of Health, Saudi Arabia approved retrospective secondary use of these data (IRB log No. 26-19-R3; 4 May 2026; expedited review).

## 3. Results

### 3.1. Survey Participation, Respondent Characteristics, and Scale Reliability

Participation increased from 180/490 (36.73%) in 2023 to 349/515 (67.77%) in 2025. In the complete functional role distribution, nursing accounted for 56/180 (31.1%) and 139/349 (39.8%), and physicians for 48/180 (26.7%) and 82/349 (23.5%). Direct patient contact increased from 69.4% to 78.5%. Arabic questionnaires accounted for 147/180 (81.7%) and 248/349 (71.1%). Table 1 includes all functional role groups. Table S2 gives the work-area distribution and the remaining adjustment characteristics. The job-position distribution with sparse categories combined is provided in Table S3.

Overall 32-item alpha was 0.904 in 2023 and 0.918 in 2025, based on 115 and 229 complete responses, respectively (Table S4). Domain coefficients ranged from 0.457 to 0.775 in 2023 and from 0.597 to 0.807 in 2025. Internal consistency was relatively low for Staffing and Work Pace and Hospital Management Support, particularly in 2023, limiting interpretation of these domain comparisons. High overall alpha does not establish a single validated construct.

### 3.2. Changes in PSC Domain Scores and Benchmarking

The overall average percent-positive score increased from 69.43% to 78.50%, a difference of 9.07 percentage points (bootstrap 95% CI 5.45–12.85). The largest point increases occurred in Communication Openness (15.62 percentage points), Hospital Management Support for Patient Safety (13.13), and Reporting Patient Safety Events (12.02). Staffing and Work Pace increased by 2.75 percentage points, but its confidence interval included no change (−2.71 to 8.26; Table 2).

**Figure 2:**
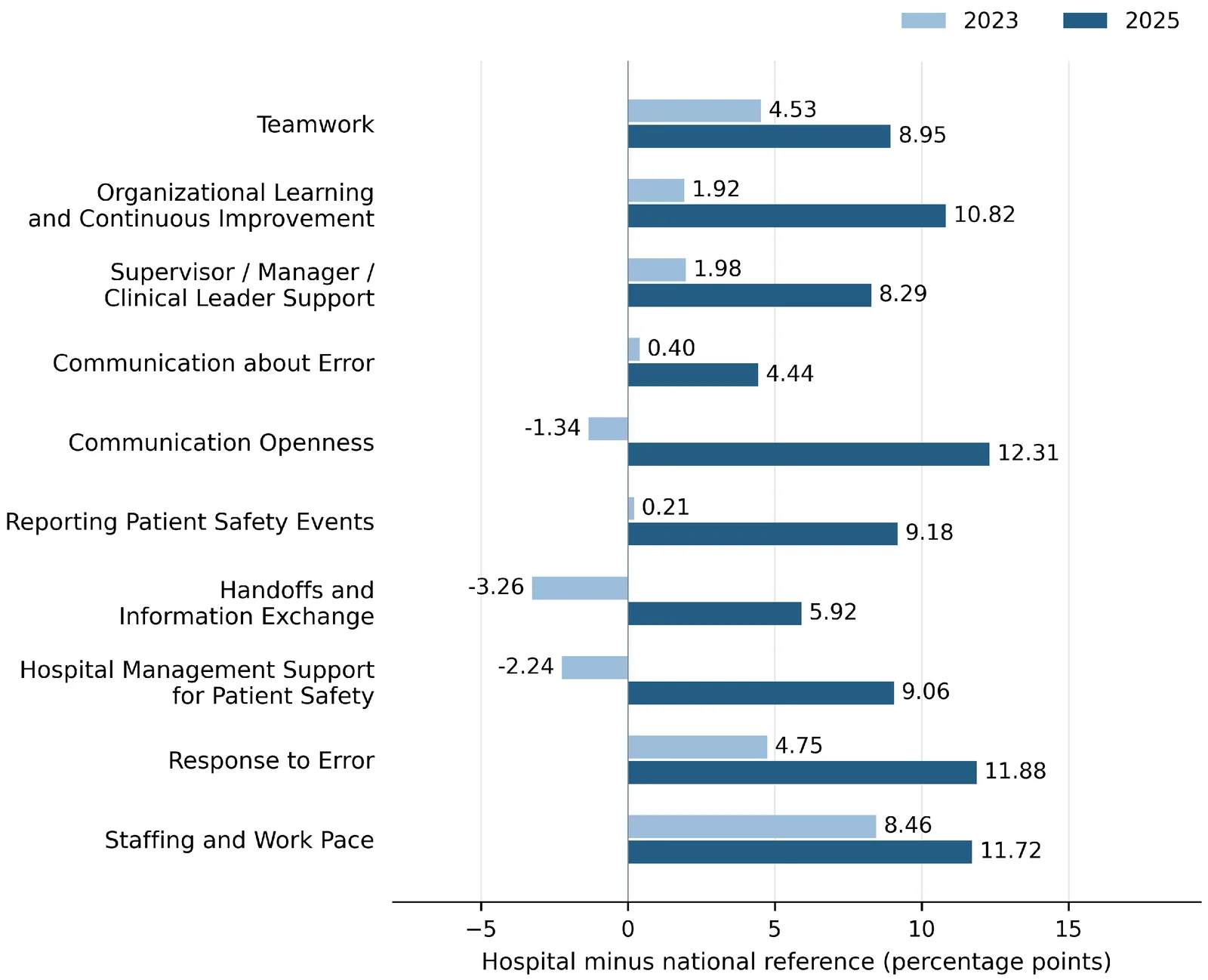
Hospital minus same-year national percent-positive scores (percentage points). Positive values favor the hospital; comparisons are descriptive.

**Figure 3:**
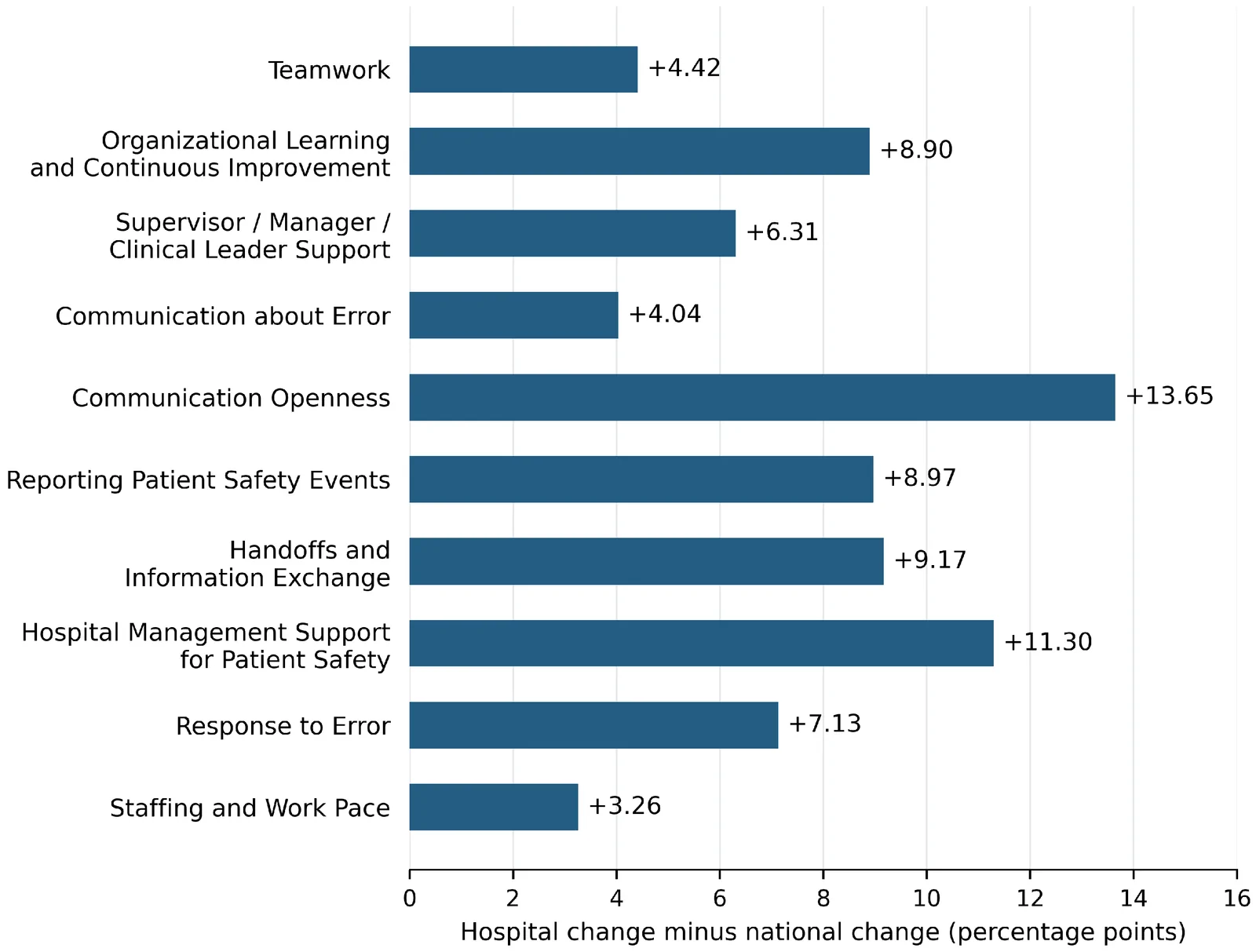
Benchmark-adjusted change, calculated as the hospital change from 2023 to 2025 minus the corresponding change in the supplied national benchmark. Domains follow the order of Figure 2, without ranking by change. These descriptive contrasts do not estimate intervention effects and have no benchmark-based confidence intervals.

The hospital’s overall average exceeded the national benchmark by 1.54 percentage points in 2023 and 9.26 in 2025. Hospital percentages were above the corresponding reference values in all ten domains in 2025. Figure 2 and Figure 3 show the descriptive benchmark gaps and changes. Figure S1 shows the hospital percent-positive profile.

The overall respondent-level summary increased from 3.814 (SD 0.479) in 2023 to 4.038 (SD 0.524) in 2025. The unadjusted difference was 0.224 points (95% CI 0.135–0.313; *p* < 0.001). After adjustment for job group, hospital tenure, weekly working hours, and direct patient contact, the difference was 0.243 points (95% CI 0.149–0.337; *p* < 0.001). Nine domain differences remained significant after Holm correction (Table 3). Staffing and Work Pace showed an inconclusive adjusted difference of 0.109 points (95% CI −0.016–0.234; *p* = 0.087).

The overall difference was similar after reweighting the 2025 sample to the exact 2023 role distribution (0.247 points; 95% CI 0.155–0.340), requiring at least half of the items within each domain (0.249; 95% CI 0.155–0.343), restricting analysis to 344 complete 32-item responses (0.237; 95% CI 0.115–0.359), and additionally adjusting for unit tenure and language (0.247; 95% CI 0.154–0.341) (Table S5). Role-reweighted domain analyses also retained the same nine positive differences after Holm correction, with Communication Openness, Reporting Patient Safety Events, and Hospital Management Support having the largest point increases (Table S6).

**Figure 4:**
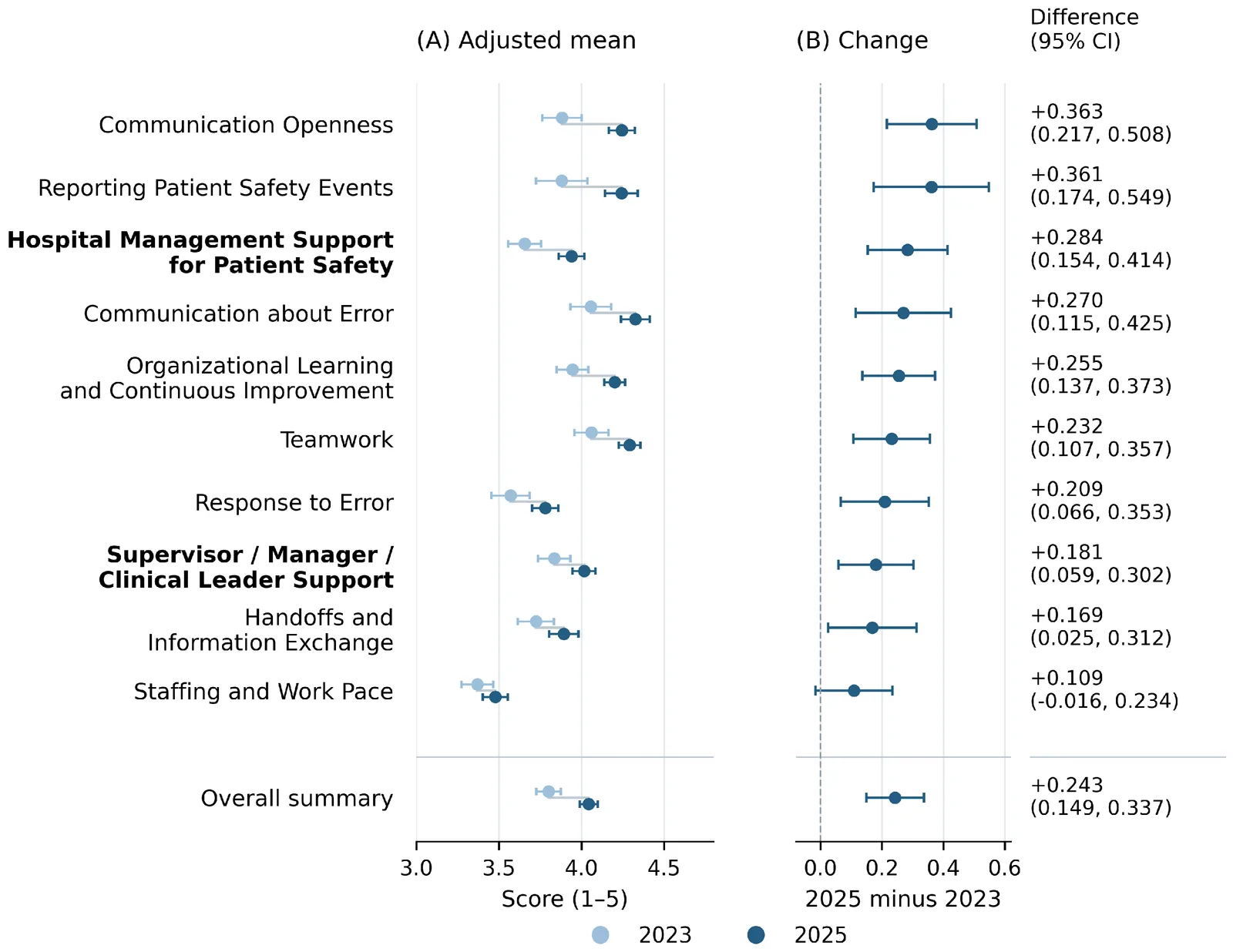
Covariate-adjusted patient safety culture scores in 2023 and 2025. (**A**) Mean scores standardized to the same pooled respondent covariate distribution within each outcome. (**B**) Adjusted differences (2025 minus 2023) with pointwise 95% confidence intervals based on heteroskedasticity-consistent HC3 standard errors. Models adjust for job group, hospital tenure, weekly working hours, and direct patient contact. Domains are ordered by adjusted change; the exploratory overall summary is separate. Bold labels identify leadership-support domains. Scores use the 1–5 scale and differ from the observed sample means in Table 3. Ranks are descriptive.

Figure 4 compares the covariate-standardized scores and adjusted differences. On the 1–5 scale, the largest adjusted point increases were in Communication Openness (0.363), Reporting Patient Safety Events (0.361), and Hospital Management Support (0.284). Supervisor/Manager/Clinical Leader Support also improved (0.181), ranking eighth by point estimate. The ordering describes point estimates and does not establish differences between domain changes.

### 3.3. Global Survey Indicators

The proportion of respondents who would recommend the hospital to family members or loved ones increased from 81.1% (146/180) in 2023 to 86.8% (303/349) in 2025 (Table 4). The distribution of patient safety grades shifted upward, with “Excellent” increasing from 33.3% (60/180) to 48.4% (169/349) and “Fair” decreasing from 5.0% (9/180) to 2.0% (7/349). The proportion reporting no patient safety events in the previous 12 months increased from 26.7% (48/180) to 35.2% (123/349), whereas the 6–10 and 11 or more categories decreased. The ordered event-reporting distribution differed between waves (Mann–Whitney *p* = 0.011). After covariate adjustment, the odds ratio for reporting no events in 2025 versus 2023 was 1.49 (95% CI 0.98–2.28; *p* = 0.065). Among nursing respondents, no-event reports increased from 9/56 (16.1%) to 44/139 (31.7%); corresponding proportions among physicians were 22/48 (45.8%) and 46/82 (56.1%). These subgroup descriptions are exploratory and do not identify reporting opportunities or exposure. Figures S2 and S3  show the safety-grade and event-reporting distributions.

Among nursing respondents with at least one year of hospital tenure, the proportion reporting no events increased from 9/56 (16.1%) in 2023 to 33/115 (28.7%) in 2025. In 2025, 11/24 (45.8%) nursing respondents with less than one year of tenure reported no events; no respondents in this tenure subgroup were present in the 2023 nursing sample (Table S7).

### 3.4. PSLWR Implementation Outputs (January–June 2025)

**Figure 5:**
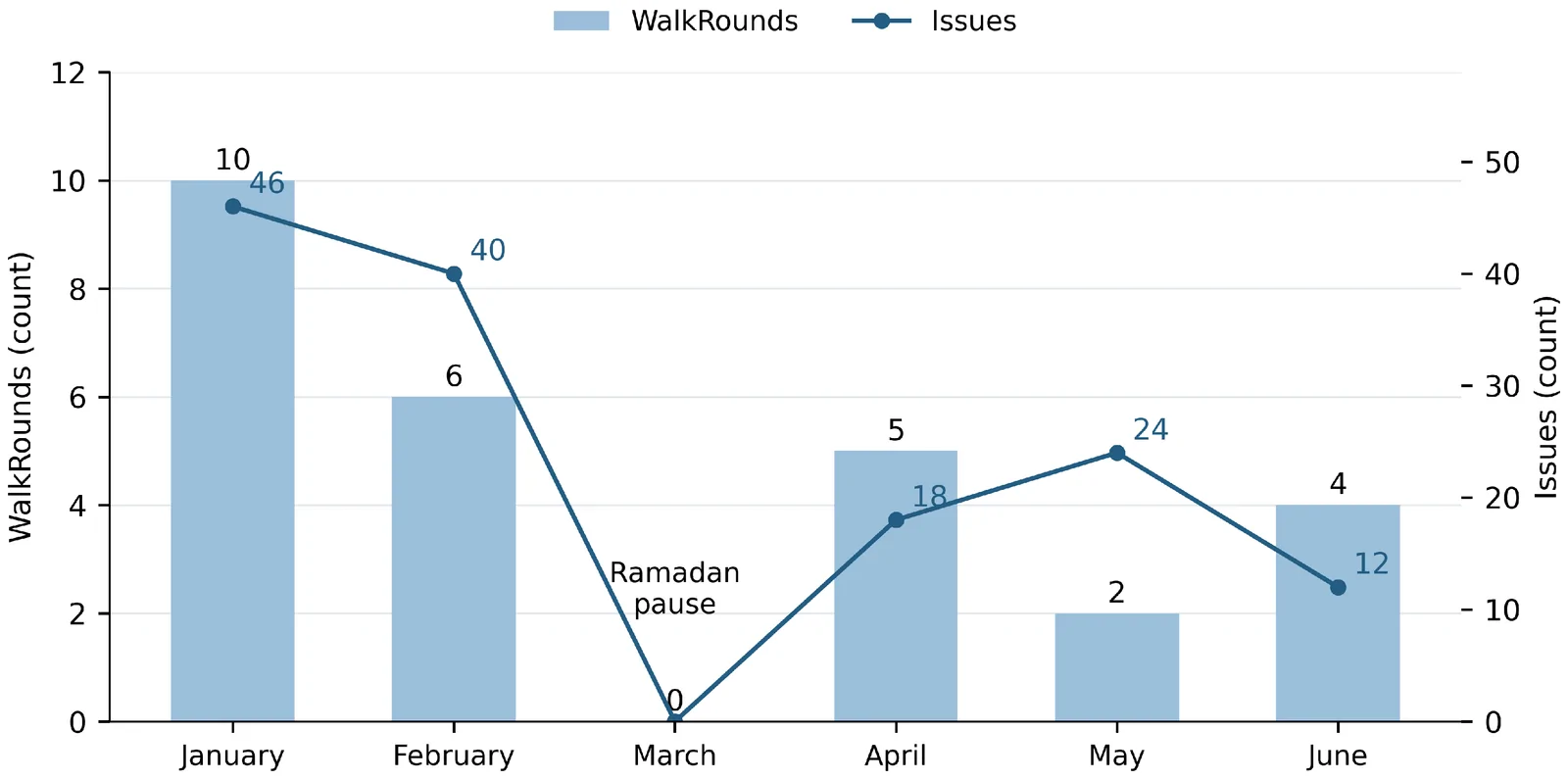
Monthly WalkRounds and identified issues, January–June 2025. Bars show rounds and the line shows issues; totals are 27 rounds and 140 issues. March was paused for Ramadan. Activity resumed in April–June at a lower intensity than in January–February.

The analyzed ledger contained 27 rounds across 16 areas, 106 staff participations, and 140 issues, averaging 3.93 participations and 5.19 issues per round. Unique staff coverage could not be determined. Monthly round counts were 10, 6, 0, 5, 2, and 4 from January to June; March was paused for Ramadan. Activity resumed thereafter, but its intensity was lower than in January–February. Group-consensus ratings were based on 27 round records (Table 5). Figure 5 shows the monthly round and issue counts.

### 3.5. Follow-Up Actions: Themes and Action Hierarchy

Of the 163 candidate recommendation units extracted from the WalkRounds reports, 51 were retained in the coded follow-up dataset (Table S1). As summarized in Table 6, these comprised 12 strong (23.5%), 32 intermediate (62.7%), and 7 weak (13.7%) actions. Staffing and Scheduling was the most frequent theme (17/51; 33.3%). Table 7 cross-tabulates primary themes and hierarchy levels. The hierarchy percentages, including the weak-action proportion, refer to this selected within-hospital corrective-action subset, not all routine monitoring or external escalations discussed during rounds. Table S8 identifies the IHI/RCA^2^ categories for the 12 recorded strong actions. Figure S4 presents the distribution of action hierarchy levels by theme.

### 3.6. Annual Incident Reporting Totals

Annual OVR totals were 1,633 in 2023, 1,731 in 2024, and 1,954 in 2025, a 19.7% increase from 2023 to 2025. These counts were not standardized for hospital activity. Figure S5 shows the annual reporting volumes.

## 4. Discussion

This facility-level evaluation found broad improvement in PSC between 2023 and 2025. The overall percent-positive score increased by 9.07 percentage points, and positive differences remained statistically significant in nine of the ten domains after covariate adjustment and Holm correction. The adjusted overall difference was 0.243 points on the 1–5 scale, with consistent findings across overall sensitivity analyses and a similar domain pattern after role reweighting. Communication Openness, Reporting Patient Safety Events, and Hospital Management Support had the largest adjusted point increases; supervisor support also improved. Communication and leadership support were therefore prominent features of the more favorable staff perceptions in 2025.

The gains in communication and leadership support are consistent with the purpose and content of the WalkRounds. Leadership-support domains assess staff perceptions of leaders’ safety priorities, responsiveness to suggestions, and action on concerns [10]. During rounds, frontline staff discussed barriers to safe practice and reporting, received clarification about OVR submission, and raised concerns for follow-up. Addressing the belief that an OVR was a complaint against a colleague created an opportunity to discuss reporting as a source of learning and improvement. Previous research highlights direct dialogue and feedback on action as relevant to perceptions of leadership engagement and safety culture [14,16,21]. These activities, together with documented responses and follow-up through department heads, offer plausible mechanisms for interpreting the observed gains. Corrective-action follow-up and feedback also formed part of the concurrent 2024–2025 risk-and-harm reduction initiative; these mechanisms cannot be attributed to WalkRounds alone.

Teamwork and Organizational Learning were already relative strengths at baseline and remained favorable in 2025. Staffing and Work Pace remained the lowest-scoring domain, with an inconclusive adjusted change. Its persistence, despite staffing and scheduling being the most frequent action theme, identifies workforce capacity and work organization as continuing priorities. Staffing concerns also recur in Saudi studies [7,8] and in earlier national research using the previous survey version [25]. The findings help distinguish areas of progress from a sustained organizational need.

This pattern has practical relevance for hospitals with constrained resources. WalkRounds and concurrent improvement initiatives operated without a dedicated implementation budget, while additional recruitment required Ministry-level decisions. Listening to staff, clarifying reporting, responding to concerns, and communicating follow-up are practices that hospital teams can incorporate into routine management. The observed gains draw attention to these locally actionable priorities alongside continued escalation of workforce needs. They suggest opportunities for improvement work within the hospital’s existing responsibilities and resources.

The action analysis adds practical information about organizational responses to frontline concerns. Fifteen of the 17 staffing and scheduling actions were classified as intermediate, while strong actions were concentrated in environment, design, and wayfinding (6 of 7) and equipment and maintenance (4 of 5). Staff deployment and team-leader coverage provide examples of intermediate responses in Table S8. The IHI hierarchy offers a framework for examining action design and identifying opportunities for stronger system responses [18,19,22]. Combining this framework with thematic analysis can help hospital teams prioritize follow-up and review recurring concerns.

Reporting indicators were mixed. Annual organizational OVR counts increased, while a larger proportion of survey respondents reported submitting no events in the preceding 12 months. Meanwhile, ratings in the Reporting Patient Safety Events domain improved. The rise in nursing respondents reporting no events (16.1% to 31.7%) was also present among those with at least one year of hospital tenure (16.1% to 28.7%), rather than being confined to very recent arrivals (Table S7). These findings distinguish perceptions of reporting practices from respondents’ own reporting activity. Discussions about reporting barriers and the announcement of recognition for frequent reporters provide relevant organizational context. They draw attention to how staff understand reportable events and how opportunities and encouragement to report may differ across groups. Reporting participation and event types deserve attention alongside total volume.

Combining routinely collected sources broadens the practical value of this evaluation. Surveys identify perceived strengths and priorities, WalkRounds records describe frontline concerns and organizational responses, and OVR totals describe reporting volume. National percentages add a descriptive reference. Together, these sources inform improvement planning by identifying gains in staff perceptions alongside concerns requiring further attention. They support sustaining communication and leadership follow-up while directing continued effort toward workforce capacity and reporting participation.

Several limitations should be considered. This retrospective, single-hospital study had no pre-intervention trend or suitable comparison group, so the contribution of WalkRounds could not be separated from concurrent initiatives and secular change. Increased participation and the observed difference in survey familiarity may have influenced the responding sample. Adjustment and reweighting address measured characteristics but cannot eliminate unmeasured selection or nonresponse bias; individual survey understanding and encouragement were not measured. Anonymous responses could not be linked across waves, and uncertainty estimates assumed independence.

Measurement limitations also affect interpretation. Low internal consistency in some domains, particularly Hospital Management Support and Staffing and Work Pace, warrants care in interpreting their changes, and the overall summary was exploratory. WalkRounds ratings were unvalidated consensus observations obtained with leaders present and were susceptible to social-desirability and ceiling effects. The first author’s operational involvement and the absence of independent duplicate coding may have influenced documentation and interpretation. Action levels describe design strength; effectiveness and sustainability were not independently assessed, and individual receipt of feedback was not measured. OVR counts covered different periods from the surveys and lacked activity denominators and event-level severity data. Counts may vary with reporting propensity, hospital activity, and event occurrence, and cannot establish changes in patient harm.

Future improvement work should build on the observed gains by maintaining dialogue with frontline staff, communicating responses to their concerns, and reviewing corrective actions for recurrent hazards. Workforce planning and work organization remain priorities alongside these practices. Repeated culture measurements, assessment of feedback receipt and sustained action outcomes, and suitable comparison designs would strengthen further evaluation. These priorities are consistent with literature emphasizing leadership engagement, feedback, and systems-oriented improvement [12,13,16,18].

## 5. Conclusions

PSC scores were higher in 2025 in nine domains after adjustment for respondent characteristics and correction for multiple testing, including hospital management and supervisor support. Staffing and Work Pace remained lowest, with an inconclusive change. WalkRounds and follow-up records documented frontline concerns and organizational responses alongside these gains. Communication, leadership responsiveness, and follow-up deserve attention within existing resources, alongside escalation of structural workforce needs. Reporting indicators were mixed; the study did not isolate WalkRounds’ contribution or establish reduced patient harm.

## Figures and Tables

**Table 1:** Survey participation and key respondent characteristics.

Characteristic	2023	2025
Reported staff denominator	490	515
Completed surveys	180	349
Response rate	36.73%	67.77%
Administrative and patient services	7 (3.9%)	15 (4.3%)
Allied health	20 (11.1%)	46 (13.2%)
Healthcare assistants	16 (8.9%)	23 (6.6%)
Management	11 (6.1%)	15 (4.3%)
Nursing	56 (31.1%)	139 (39.8%)
Pharmacy	16 (8.9%)	19 (5.4%)
Physicians	48 (26.7%)	82 (23.5%)
Quality and infection control	6 (3.3%)	10 (2.9%)

Note. Reported response rates use the staff counts supplied for the Saudi Patient Safety Center (SPSC) facility surveys as denominators; job-group percentages use completed survey responses. Percentages may not sum to 100% because of rounding.

**Table 2:** Hospital percent-positive scores and change with descriptive national reference values.

Domain	Hospital 2023%	Hospital 2025%	Change pp	95% CI	National 2023%	National 2025%
Overall	69.43	78.50	9.07	5.45 to 12.85	67.89	69.24
Teamwork	84.55	89.48	4.93	0.46 to 9.51	80.02	80.53
Organizational Learning	79.46	89.75	10.29	5.45 to 15.27	77.54	78.93
Supervisor Support	73.81	81.16	7.35	2.01 to 12.79	71.83	72.87
Communication about Error	74.26	80.98	6.72	0.64 to 13.05	73.86	76.54
Communication Openness	65.01	80.63	15.62	9.67 to 21.48	66.35	68.32
Reporting Patient Safety Events	68.37	80.39	12.02	4.74 to 19.41	68.16	71.21
Handoffs and Information Exchange	67.44	77.25	9.80	2.87 to 16.87	70.70	71.33
Hospital Management Support	62.79	75.92	13.13	7.30 to 19.11	65.03	66.86
Response to Error	61.52	69.57	8.05	1.90 to 14.26	56.77	57.69
Staffing and Work Pace	57.09	59.84	2.75	−2.71 to 8.26	48.63	48.12

Note. pp = percentage points; CI = pointwise 95% percentile bootstrap confidence interval based on 10,000 within-wave respondent resamples. Change is 2025 minus 2023, in percentage points, calculated before rounding. National columns reproduce same-year portal reference values; national uncertainty estimates were unavailable.

**Table 3:** Respondent-level scores and adjusted differences on the 1–5 scale.

Outcome	2023	2025	Adjusted Difference 95% CI	*p*	Holm *p*
Overall	3.81 ± 0.48 *n* = 180	4.04 ± 0.52 *n* = 349	0.243 (0.149 to 0.337)	<0.001	—
Teamwork	4.06 ± 0.65 *n* = 180	4.29 ± 0.62 *n* = 349	0.232 (0.107 to 0.357)	<0.001	0.002
Organizational Learning	3.95 ± 0.63 *n* = 180	4.20 ± 0.60 *n* = 348	0.255 (0.137 to 0.373)	<0.001	<0.001
Supervisor Support	3.84 ± 0.64 *n* = 179	4.01 ± 0.67 *n* = 348	0.181 (0.059 to 0.302)	0.004	0.015
Communication about Error	4.08 ± 0.81 *n* = 179	4.31 ± 0.82 *n* = 346	0.270 (0.115 to 0.425)	<0.001	0.003
Communication Openness	3.89 ± 0.78 *n* = 179	4.24 ± 0.73 *n* = 346	0.363 (0.217 to 0.508)	<0.001	<0.001
Reporting Patient Safety Events	3.90 ± 1.02 *n* = 171	4.23 ± 0.91 *n* = 338	0.361 (0.174 to 0.549)	<0.001	0.001
Handoffs and Information Exchange	3.72 ± 0.72 *n* = 159	3.89 ± 0.77 *n* = 309	0.169 (0.025 to 0.312)	0.021	0.043
Hospital Management Support	3.67 ± 0.66 *n* = 178	3.93 ± 0.73 *n* = 348	0.284 (0.154 to 0.414)	<0.001	<0.001
Response to Error	3.59 ± 0.75 *n* = 179	3.77 ± 0.78 *n* = 348	0.209 (0.066 to 0.353)	0.004	0.015
Staffing and Work Pace	3.40 ± 0.66 *n* = 179	3.46 ± 0.77 *n* = 349	0.109 (−0.016 to 0.234)	0.087	0.087

Note. SD = standard deviation; CI = confidence interval. Mean ± SD and available-case n are shown for each wave. Regression adjusts for functional job group, hospital tenure, weekly working hours, and direct patient contact; 95% CIs use HC3 standard errors. Holm *p* values adjust the ten domain tests. Overall is an exploratory equal-weight mean of available domain means, assessed separately. Unadjusted overall Welch difference = 0.224 (95% CI 0.135–0.313; *p* < 0.001).

**Table 4:** Global survey indicators in 2023 and 2025.

Indicator/Category	2023	2025	Change (pp)
Would recommend hospital to family/loved ones			
Yes	146 (81.1%)	303 (86.8%)	+5.71
No	34 (18.9%)	46 (13.2%)	−5.71
Patient safety grade			
Poor	1 (0.6%)	1 (0.3%)	−0.27
Fair	9 (5.0%)	7 (2.0%)	−2.99
Good	39 (21.7%)	57 (16.3%)	−5.33
Very Good	71 (39.4%)	115 (33.0%)	−6.49
Excellent	60 (33.3%)	169 (48.4%)	+15.09
Events reported in past 12 months			
None	48 (26.7%)	123 (35.2%)	+8.58
1 to 2	48 (26.7%)	100 (28.7%)	+1.99
3 to 5	34 (18.9%)	59 (16.9%)	−1.98
6 to 10	26 (14.4%)	30 (8.6%)	−5.85
11 or more	24 (13.3%)	37 (10.6%)	−2.73

Note. pp = percentage points. Change represents the 2025 percentage minus the 2023 percentage for the same response category.

**Table 5:** PSLWR implementation summary (January–June 2025).

Indicator	Value
WalkRounds conducted	27
Departments/areas covered	16
Staff participations	106
Issues identified (total)	140
Mean staff participations per WalkRound	3.93
Mean issues identified per WalkRound	5.19
Reporting confidence (1–5)	4.63 ± 0.69
Leadership involvement observed (1–5)	4.07 ± 0.92
Improvements witnessed in last quarter (1–5)	4.44 ± 0.70
Staff involvement in improving quality (1–5)	4.70 ± 0.47

Note. Each rating is one frontline group-consensus score obtained with leaders present, not an independent staff response. Values are mean ± SD across 27 round records. Staff participations may include repeat attendance.

**Table 6:** Action hierarchy and primary theme summary for documented follow-up actions.

Section	Category	Count	Percent
Action hierarchy	Strong	12	23.5%
Action hierarchy	Intermediate	32	62.7%
Action hierarchy	Weak	7	13.7%
Primary theme	Staffing & Scheduling	17	33.3%
Primary theme	Environment/Design/Wayfinding	7	13.7%
Primary theme	Communication & Coordination	6	11.8%
Primary theme	Equipment/Maintenance	5	9.8%
Primary theme	Other	5	9.8%
Primary theme	Policy/Protocol/Standard Work	5	9.8%
Primary theme	Training & Education	3	5.9%
Primary theme	IT/Digital Systems	2	3.9%
Primary theme	Supplies/Stock	1	2.0%

Note. Percentages are based on 51 eligible documented follow-up actions. “Other” included documented actions that did not fit clearly into the predefined operational themes.

**Table 7:** Primary theme by action hierarchy (counts).

Primary theme	Strong	Intermediate	Weak	Total
Staffing & Scheduling	1	15	1	17
Environment/Design/Wayfinding	6	1	0	7
Communication & Coordination	0	3	3	6
Equipment/Maintenance	4	1	0	5
Other	0	5	0	5
Policy/Protocol/Standard Work	1	4	0	5
Training & Education	0	1	2	3
IT/Digital Systems	0	1	1	2
Supplies/Stock	0	1	0	1

Note. Counts reflect eligible documented follow-up actions coded by primary theme and action hierarchy.

## Data Availability

The data used in this study are not publicly available because they are based on internal organizational datasets and may be shared only with institutional permission.

## Supplementary Materials

The following supporting information can be downloaded at: https://www.mdpi.com/article/10.3390/healthcare14213704/s1, Table S1: Recommendation units and primary reasons for exclusion from the coded follow-up dataset; Table S2: Work areas and adjustment characteristics; Table S3: Respondent job-position distribution with sparse categories combined; Table S4: Internal consistency and complete-case counts; Table S5: Overall score sensitivity analyses; Table S6: Domain differences after reweighting the 2025 respondents to the exact 2023 job-position distribution; Table S7: Nursing respondents reporting no patient safety events, by hospital tenure; Table S8: The 12 recorded strong actions and illustrative intermediate and weak actions, with corresponding IHI/RCA^2^ categories; Figure S1: Hospital percent-positive scores by domain in 2023 and 2025. Values are observed scores and are distinct from the covariate-adjusted means in main Figure 4; Figure S2: Patient safety grade distribution among survey respondents in 2023 and 2025. Bars and labels show category-specific percentages; Figure S3: Self-reported number of patient safety events in the previous 12 months in 2023 and 2025. Bars and labels show category-specific percentages; Figure S4: Recorded action hierarchy by primary theme among the 51 analyzed follow-up actions. Segments are ordered strong, intermediate, and weak; totals are shown at the end of each bar. Theme order matches Table 7; Figure S5: Annual occurrence variance report (OVR) totals, 2023–2025. Counts are not standardized for hospital activity and do not measure patient harm.

## Abbreviations

The following abbreviations are used in this manuscript:

**Abbreviation**	**Meaning**
AHRQ	Agency for Healthcare Research and Quality
IHI	Institute for Healthcare Improvement
IRB	Institutional Review Board
OVR	Occurrence variance report
PSC	Patient safety culture
PSLWR	Patient Safety Leadership WalkRounds
RCA^2^	Root Cause Analyses and Actions
SOPS	Surveys on Patient Safety Culture
SPSC	Saudi Patient Safety Center
VA	US Department of Veterans Affairs
