# Supplementary material for: Changes in Patient Safety Culture Alongside Leadership WalkRounds in Shaqra General Hospital from 2023 to 2025
Source: Healthcare (Basel). 2026 Nov 1;14(21):3704. doi: 10.3390/healthcare14213704 (PMC13763178; doi:10.3390/healthcare14213704)
Supplement: Supplementary file 1 [file healthcare-14-03704-s001.zip › healthcare-4628885-supplementary.pdf]

Supplementary materials

Changes in Patient Safety Culture Alongside Leadership WalkRounds in Shaqra General Hospital from 2023 to 2025

Mahah Saad Almohanna and Abeer Ghazi Alharbi

Tables S1–S8 and Figures S1–S5 accompany the manuscript. All values are aggregate summaries; no individual survey responses or identifiable organizational records are reproduced.

Table S1. Recommendation units and primary reasons for exclusion from the coded follow-up dataset.

Stage or primary reason	Units, n	Definition
Extracted candidate recommendation units	163	Report units screened for a specified corrective action, distinct from the separate count of 140 safety issues.
1. Duplicate or overlapping recommendations	4	Repeated recommendations or overlap with another action.
2. Units without a specified corrective action	77	Includes lessons learned, previously resolved problems, and explanatory rationale.
3. Escalation outside hospital implementation authority	3	Actions requiring higher-authority action and not implementable within the hospital.
4. Follow-up or monitoring of existing practice without a new corrective action	28	Monitoring or reinforcement of an existing policy, protocol, or practice only.
Total excluded units	112	Sum of the four primary reasons.
Retained coded follow-up actions	51	Departmental action dataset used for the thematic and hierarchy summaries.

Note. Counts summarize the recommendation units using report content and the department’s operational clarification. Each excluded unit is counted under one primary reason. The 140 safety issues form a separate issue count; one issue could generate more than one recommendation unit.

healthcare

MDPI

Supplementary materials

Table S2. Work areas and adjustment characteristics.

Variable and category	2023 *n* (%)	2025 *n* (%)
Multiple hospital units or no specific unit	14 (7.8%)	17 (4.9%)
Labor & Delivery, Obstetrics & Gynecology	14 (7.8%)	23 (6.6%)
Pediatrics (including NICU, PICU)	14 (7.8%)	26 (7.4%)
Outpatient department (OPD)	10 (5.6%)	22 (6.3%)
Preoperative, operating room, post-anesthesia, or perioperative care	0 (0.0%)	10 (2.9%)
Laboratory	13 (7.2%)	17 (4.9%)
Pharmaceutical care services	11 (6.1%)	9 (2.6%)
Radiology, Imaging	5 (2.8%)	10 (2.9%)
Medical Unit	9 (5.0%)	7 (2.0%)
Quality, Risk management, Patient safety, Clinical audit, Performance improvement	10 (5.6%)	10 (2.9%)
Medical affairs (Clinical-Technical)	0 (0.0%)	11 (3.2%)
Other specified work areas	33 (18.3%)	36 (10.3%)
Surgical Unit	10 (5.6%)	13 (3.7%)
Emergency departments	5 (2.8%)	39 (11.2%)
ICU (All Adult Types)	6 (3.3%)	15 (4.3%)
Other work areas combined	26 (14.4%)	84 (24.1%)
Hospital tenure: Less than 1 year	8 (4.4%)	37 (10.6%)
Hospital tenure: 1 to 5 years	92 (51.1%)	148 (42.4%)
Hospital tenure: 6 to 10 years	32 (17.8%)	79 (22.6%)
Hospital tenure: 11 or more years	48 (26.7%)	85 (24.4%)
Unit tenure: Less than 1 year	13 (7.2%)	47 (13.5%)
Unit tenure: 1 to 5 years	100 (55.6%)	171 (49.0%)
Unit tenure: 6 to 10 years	36 (20.0%)	74 (21.2%)
Unit tenure: 11 or more years	31 (17.2%)	57 (16.3%)
Weekly working hours: Less than 30	8 (4.4%)	5 (1.4%)
Weekly working hours: 30–40	69 (38.3%)	122 (35.0%)
Weekly working hours: More than 40	103 (57.2%)	222 (63.6%)
Direct patient contact: Yes	125 (69.4%)	274 (78.5%)
Direct patient contact: No	55 (30.6%)	75 (21.5%)
Survey language: Arabic	147 (81.7%)	248 (71.1%)
Survey language: English	33 (18.3%)	101 (28.9%)

Role mapping used in adjustment: Nursing codes 1–5; Physicians 6–9; Allied health 10, 12–16, 18; Pharmacy 11; Healthcare assistants 19; Management 20–21; Quality and infection control 17, 22; Administrative and patient services 23–24. Work areas with 1–4 respondents in either wave are combined for publication; no respondents are excluded. Grouping for publication

does not change the internally analyzed survey records. NICU = neonatal intensive care unit; PICU = pediatric intensive care unit; ICU = intensive care unit; OPD = outpatient department.

healthcare

MDPI

Supplementary materials

Table S3. Respondent job-position distribution with sparse categories combined.

Job position	2023 *n* (%)	2025 *n* (%)
Nurse specialist and senior nursing roles	38 (21.1)	89 (25.5)
Other nursing roles	18 (10.0)	50 (14.3)
Resident physicians	11 (6.1)	24 (6.9)
Specialist and senior specialist physicians	26 (14.4)	50 (14.3)
Consultant physicians	11 (6.1)	8 (2.3)
Other allied health roles	7 (3.9)	19 (5.4)
Clinical technologists	13 (7.2)	27 (7.7)
Pharmacists	16 (8.9)	19 (5.4)
Healthcare assistants	16 (8.9)	23 (6.6)
Management and supervision	11 (6.1)	15 (4.3)
Quality and infection control	6 (3.3)	10 (2.9)
Administrative and patient services	7 (3.9)	15 (4.3)

Note. All 180 and 349 respondents are accounted for. Sparse job positions are combined into broader functional categories for confidentiality; individual positions with small counts are not separately reported. The eight functional groups used in regression remain those shown in Table 1.

Table S4. Internal consistency and complete-case counts.

Domain	Items	2023 *n*	2023 α	2025 *n*	2025 α
Teamwork	3	178	0.613	342	0.643
Organizational Learning	3	172	0.591	339	0.696
Supervisor Support	3	172	0.570	336	0.632
Communication about Error	3	172	0.775	337	0.807
Communication Openness	4	149	0.653	300	0.688
Reporting Patient Safety Events	2	164	0.741	325	0.690
Handoffs and Information Exchange	3	148	0.696	287	0.715
Hospital Management Support	3	161	0.474	326	0.597
Response to Error	4	166	0.741	329	0.774
Staffing and Work Pace	4	163	0.457	322	0.598
Overall 32-item scale	32	115	0.904	229	0.918

Note. Complete cases are defined separately within each domain. The two-item Reporting domain had inter-item r = 0.589 and 0.526, and Spearman–Brown coefficients = 0.741 and 0.690, in 2023 and 2025, respectively. High full-scale alpha does not validate a unidimensional total score.

Table S5. Overall score sensitivity analyses.

Analysis	N	Difference and 95% CI	*p*
2025 reweighted to exact 2023 role mix	526	0.247 (0.155 to 0.340)	< 0.001
At least half the items per domain	529	0.249 (0.155 to 0.343)	< 0.001
Complete 32-item responses	344	0.237 (0.115 to 0.359)	< 0.001
Additional unit tenure and language	529	0.247 (0.154 to 0.341)	< 0.001

CI = confidence interval. Role weights target the observed 2023 exact-role proportions. Three 2025 respondents in roles absent in 2023 received zero weight. Maximum weight = 2.666; 2025 effective sample size = 299.7. Weighted intervals condition on the estimated weights.

Table S6. Domain differences after reweighting the 2025 respondents to the exact 2023 job-position distribution.

Domain	N	Difference (95% CI)	*p*	Holm *p*
Teamwork	526	0.244 (0.125 to 0.363)	< 0.001	< 0.001
Organizational Learning	525	0.251 (0.135 to 0.366)	< 0.001	< 0.001
Supervisor Support	524	0.188 (0.065 to 0.310)	0.003	0.011
Communication about Error	522	0.247 (0.096 to 0.399)	0.001	0.007
Communication Openness	522	0.362 (0.222 to 0.502)	< 0.001	< 0.001
Reporting Patient Safety Events	506	0.333 (0.147 to 0.519)	< 0.001	0.003
Handoffs and Information Exchange	465	0.204 (0.062 to 0.347)	0.005	0.012
Hospital Management Support	523	0.295 (0.165 to 0.424)	< 0.001	< 0.001
Response to Error	524	0.207 (0.066 to 0.347)	0.004	0.012
Staffing and Work Pace	525	0.122 (-0.008 to 0.252)	0.065	0.065

Note. Scores are on the 1–5 scale. Positive differences favor 2025. Weights target the observed 2023 exact-role proportions; roles absent in 2023 receive zero weight in 2025. Models contain survey year only and use HC3 confidence intervals conditional on the estimated weights. N includes respondents with a scored outcome and positive weight. Holm correction covers the ten domain tests. This sensitivity analysis differs from the covariate-adjusted primary model in Table 3.

Table S7. Nursing respondents reporting no patient safety events, by hospital tenure.

Hospital tenure	2023 respondents, n	2023 no events, n (%)	2025 respondents, n	2025 no events, n (%)
All nursing respondents	56	9 (16.1)	139	44 (31.7)
Less than one year	0	Not estimable	24	11 (45.8)
At least one year	56	9 (16.1)	115	33 (28.7)

Note. Nursing comprises job-position codes 1–5. The outcome is the survey item on events reported during the preceding 12 months. Percentages use the number of nursing respondents in each tenure subgroup, not event counts. The 2023 less-than-one-year percentage is not estimable because its denominator is zero. These are descriptive comparisons of anonymous independent cross-sections, not changes within individual nurses.

Table S8. The 12 recorded strong actions and illustrative intermediate and weak actions, with corresponding IHI/RCA² categories.

Recorded level	Action unit and recommended response	IHI/RCA² category	Basis for category
Strong	AH-031. Open a second OB/GYN clinic using available ultrasound and computer resources, with appointment redistribution.	Tangible involvement by leadership	Allocating available resources to additional clinic capacity is a concrete leadership response to an identified service need.
Strong	AH-012. Consider redesigning the pre-triage waiting zone and adding seating with color-coded routing signs.	Architectural/physical plant changes	The physical waiting-zone and seating redesign is the relevant mechanism; signs alone do not establish an architectural change.
Strong	AH-016. Consider unifying emergency care access through one main gate.	Simplify process	A unified entry route reduces competing routes into emergency care and simplifies patient navigation.
Strong	AH-058. Install push handles on emergency exit doors to permit opening from inside.	Architectural/physical plant changes	Changing exit-door hardware addresses access through a physical-environment modification.
Strong	AH-066. Consider expanding or renovating physiotherapy space and optimizing its use.	Architectural/physical plant changes	The proposed expansion or renovation addresses restricted treatment space through changes to the physical environment.
Strong	AH-143. Expand the outpatient department.	Architectural/physical plant changes	Additional outpatient space addresses crowding through a physical-capacity response.
Strong	AH-147. Identify needed furniture and equipment to replace materials that do not meet safety and infection-control requirements.	Tangible involvement by leadership	The replacement-resource component is the relevant mechanism. Inventory checking by itself is not the basis for a strong classification.
Strong	AH-002. Replace old or damaged wheelchairs after equipment review.	Tangible involvement by leadership	Procurement and provision of replacement wheelchairs address a safety-related resource need. The category does not imply device redesign or usability testing.
Strong	AH-047. Consider requesting and providing a ward ultrasound machine to reduce reliance on the delivery room.	Tangible involvement by leadership	The equipment-provision component targets a resource dependency through leadership allocation of needed equipment.
Strong	AH-050. Request ten additional infusion and syringe pumps to meet demand.	Tangible involvement by leadership	The intended provision of additional pumps addresses equipment availability through a resource decision; the request itself does not establish delivery.

Recorded level	Action unit and recommended response	IHI/RCA² category	Basis for category
Strong	AH-063. Invest in adjustable and mobile treatment beds suitable for physiotherapy.	Tangible involvement by leadership	Investment in equipment suited to treatment needs is resource provision. No new-device usability testing is asserted.
Strong	AH-036. Implement a mandatory electronic triage step before opening a patient file.	Engineering control (forcing function)	The stated mechanism is a required prerequisite that prevents progression without triage, rather than a reminder or overridable alert.
Intermediate	AH-035. Redistribute nursing staff and assess the required nurse-to-patient ratio.	Increase in staffing/decrease in workload	Staff redistribution changes coverage and workload allocation without requiring a physical or technical constraint.
Intermediate	AH-115. Assign a team leader to each emergency area instead of relying on one leader across areas.	Increase in staffing/decrease in workload	Additional area-level coordination redistributes supervisory workload and improves coverage.
Weak	AH-076. Train staff on storage of light-sensitive medications.	Training	Conventional instruction relies on staff retaining and applying the information; no simulation-based training or system constraint is specified.

Note. IHI = Institute for Healthcare Improvement; RCA² = Root Cause Analyses and Actions. Action wording is condensed from the extracted recommendation units; AH identifiers link to the source extraction. All 12 recorded strong actions are shown, followed by illustrative intermediate and weak actions. Category labels follow the RCA² Action Hierarchy (version 2, January 2016, Figure 3), linked from the IHI tool cited as reference 19 in the main manuscript. Wording such as "consider" or "request" is retained where present. Hierarchy levels describe the recommended response mechanism, not verification of implementation or effectiveness. Replacement wheelchairs were procured and made available.

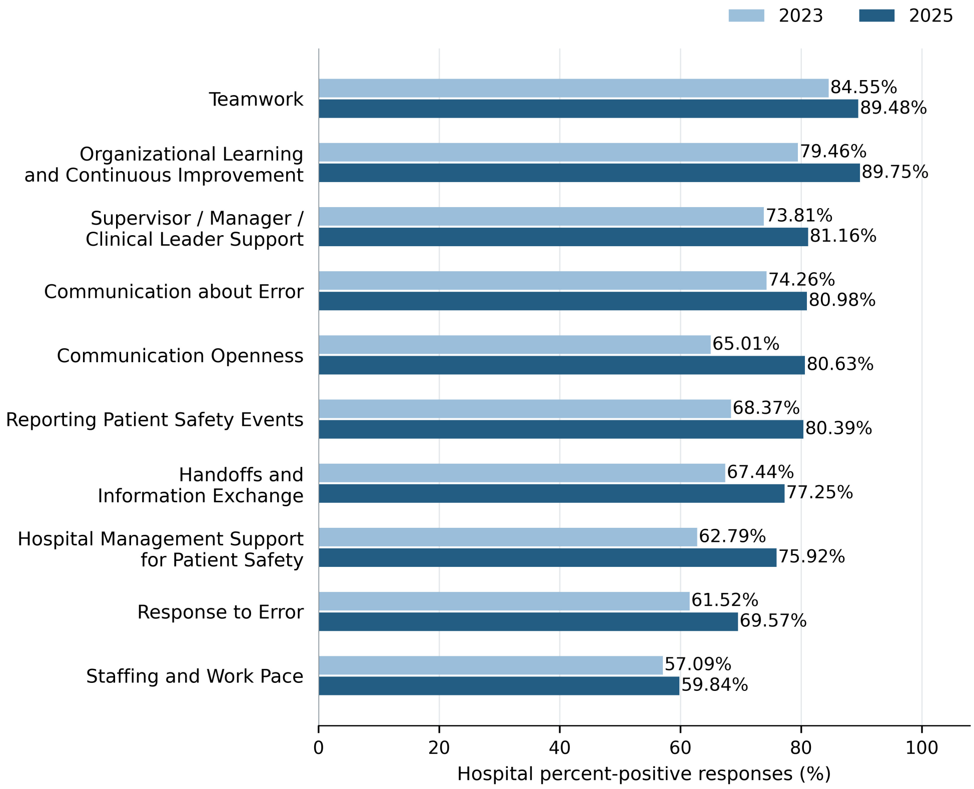

Figure S1. Hospital percent-positive scores by domain in 2023 and 2025. Values are observed scores and are distinct from the covariate-adjusted means in main Figure 4.

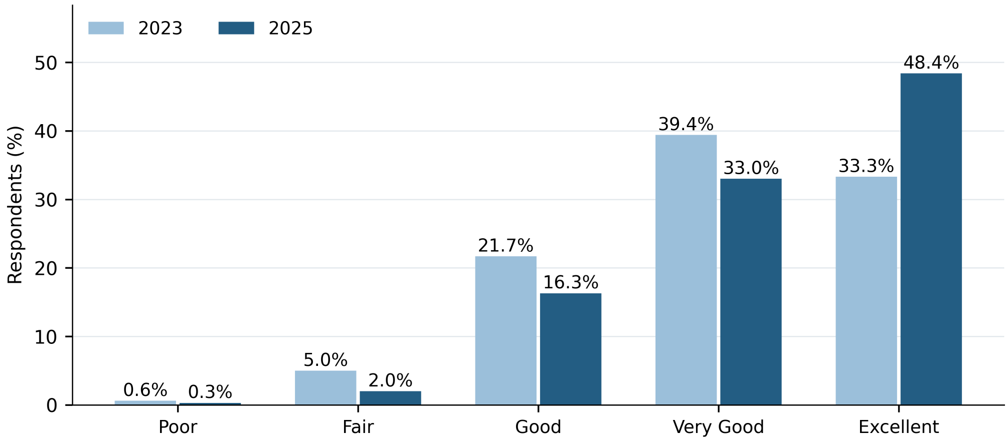

Figure S2. Patient safety grade distribution among survey respondents in 2023 and 2025. Bars and labels show category-specific percentages.

healthcare

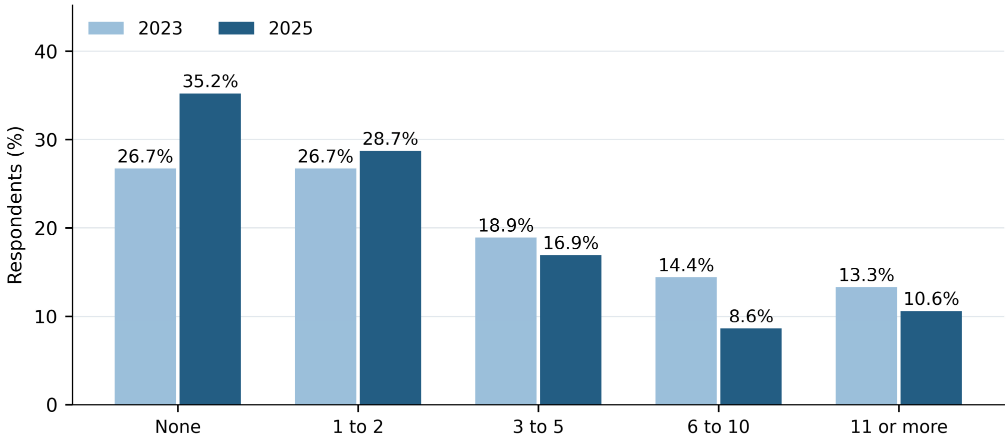

Figure S3. Self-reported number of patient safety events in the previous 12 months in 2023 and 2025. Bars and labels show category-specific percentages.

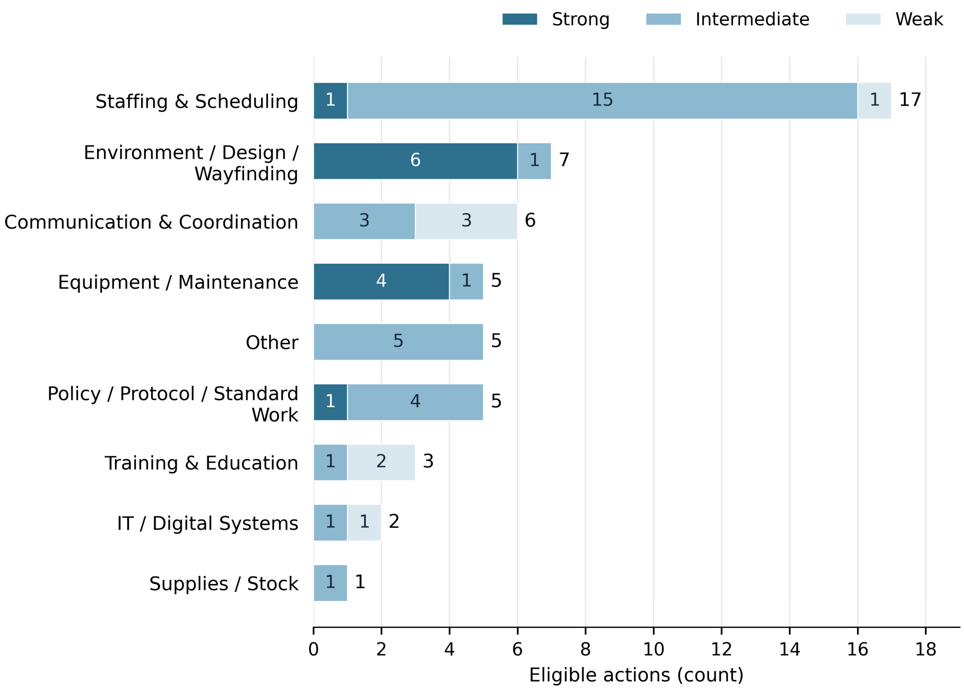

Figure S4. Recorded action hierarchy by primary theme among the 51 analyzed follow-up actions. Segments are ordered strong, intermediate, and weak; totals are shown at the end of each bar. Theme order matches Table 7.

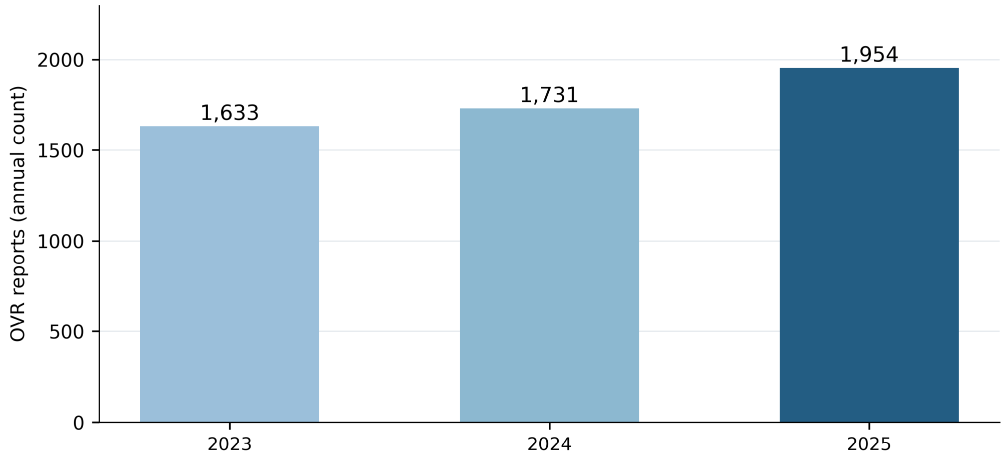

Figure S5. Annual occurrence variance report (OVR) totals, 2023–2025. Counts are not standardized for hospital activity and do not measure patient harm.
